# Supplementary material for: Whole proteome analysis of human tankyrase knockout cells reveals targets of tankyrase-mediated degradation
Source: Nat Commun. 2017 Dec 20;8:2214. doi: 10.1038/s41467-017-02363-w (PMC5738441; doi:10.1038/s41467-017-02363-w)
Supplement: Supplementary file 1 — Supplementary Information [file 41467_2017_2363_MOESM1_ESM.pdf]

Supplementary Figure 1

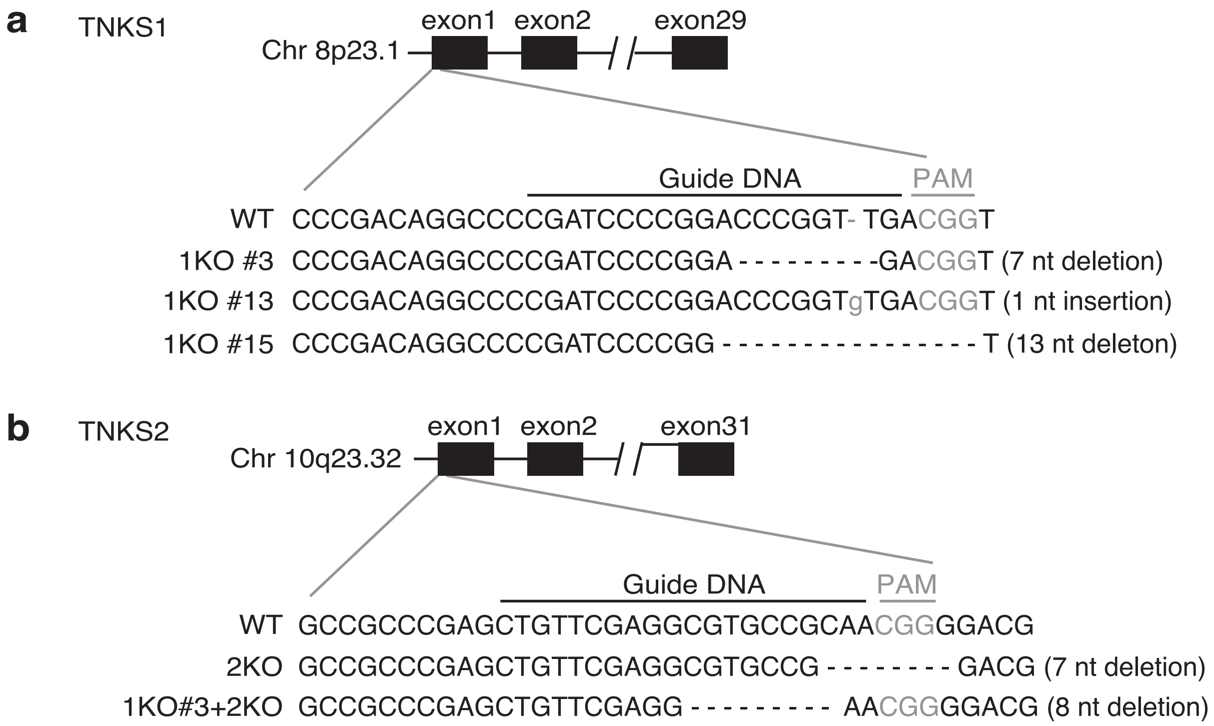

Supplementary Figure 1. Strategy for generation of TNKS KO cell lines. Schematic diagram of the exon organization of the (a) *TNKS1* or (b) *TNKS2* genes. The WT allele is indicated with the deletion or insertion indicated below it.

Supplementary Figure 2

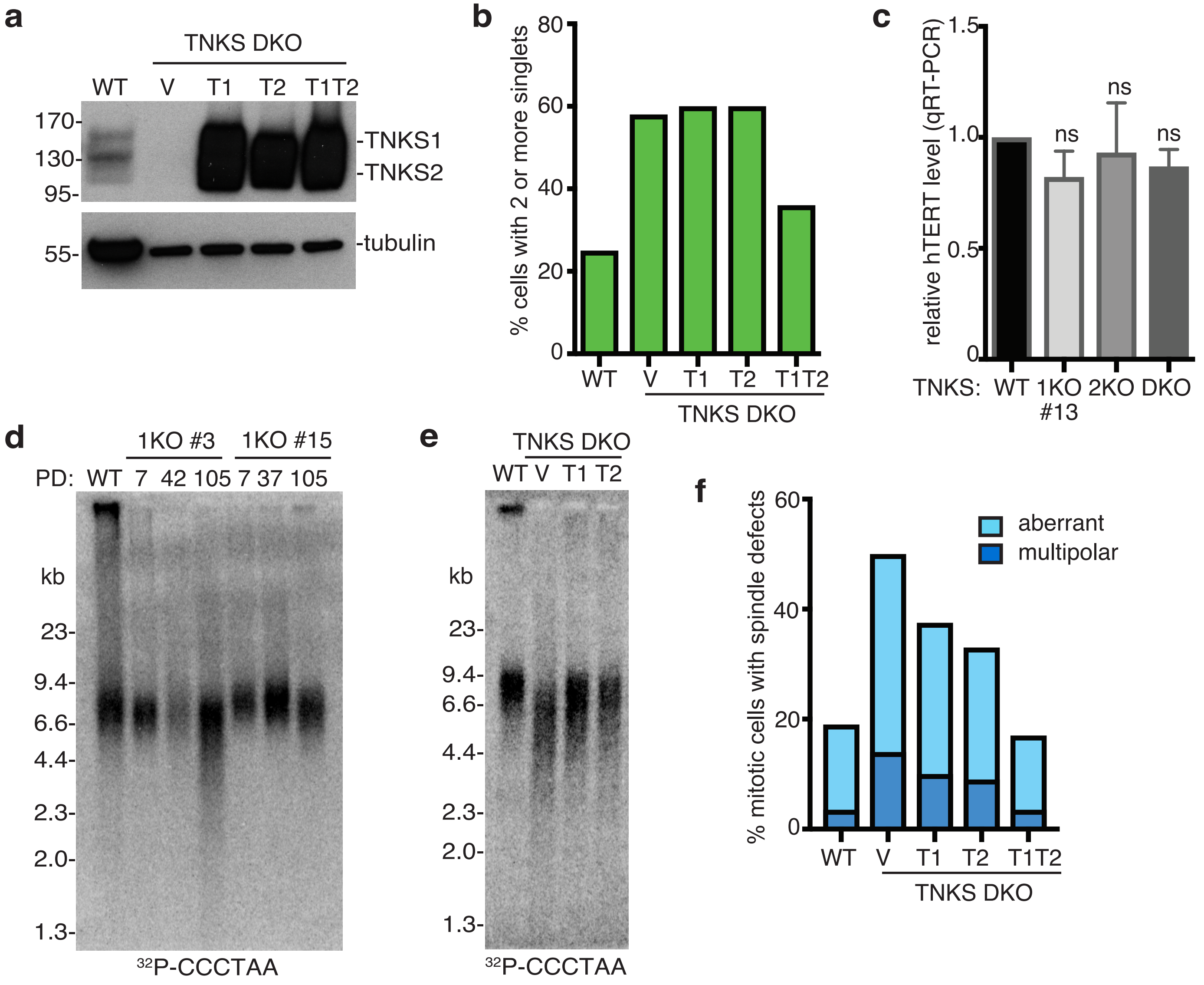

Supplementary Figure 2. Functional analysis of TNKS KO cell lines.
(a) Immunoblot analysis of WT and DKO HEK293T cells stably expressing V, T1, T2, or T1T2 probed with anti-tankyrase or anti-tubulin antibodies. (b) Both tankyrase 1 and 2 are required to rescue persistent telomere cohesion. Quantification of the frequency of mitotic cells with cohered telomeres based on FISH analysis with a 16p telo probe of mitotic WT or DKO cells stably expressing V, T1, T2, or T1T2. (n=38-75 cells each). (c) Quantification of hTERT mRNA levels using qRT-PCR. Average of two independent experiments (with 3 technical replicates each) ± SD. ns; not significant, students unpaired t-test. (d) Telomere length is maintained in TNKS1 KO cells. Analysis of telomere restriction fragments isolated from HEK293T WT, 1KO #3, or 1KO #15 cells at population doubling (PD) 7, 42 or 37, or 105, fractionated on agarose gel, denatured and hybridized with a ^{32}P-[CCCATT]$_3$ probe. (e) Either tankyrase 1 or 2 can rescue telomere shortening in DKO cells. Analysis of telomere restriction fragments isolated from WT or TNKS DKO cells stably expressing vector (V), TNKS1 (T1), or TNKS2 (T2), fractionated on agarose gel, denatured and hybridized with a ^{32}P-[CCCATT]$_3$ probe. Lentiviruses expressing V, T1 or T2 were introduce into DKO cells at PD120, harvested for analysis at PD152-154. (f) Both tankyrase 1 and 2 are required to rescue spindle defects. Quantification of the frequency of cells with aberrant mitotic spindles based on immunofluorescence analysis with β-tubulin antibody of mitotic WT or DKO cells stably expressing vector V, T1, T2, or T1T2. (n=58 mitotic cells each).

Supplementary Figure 3

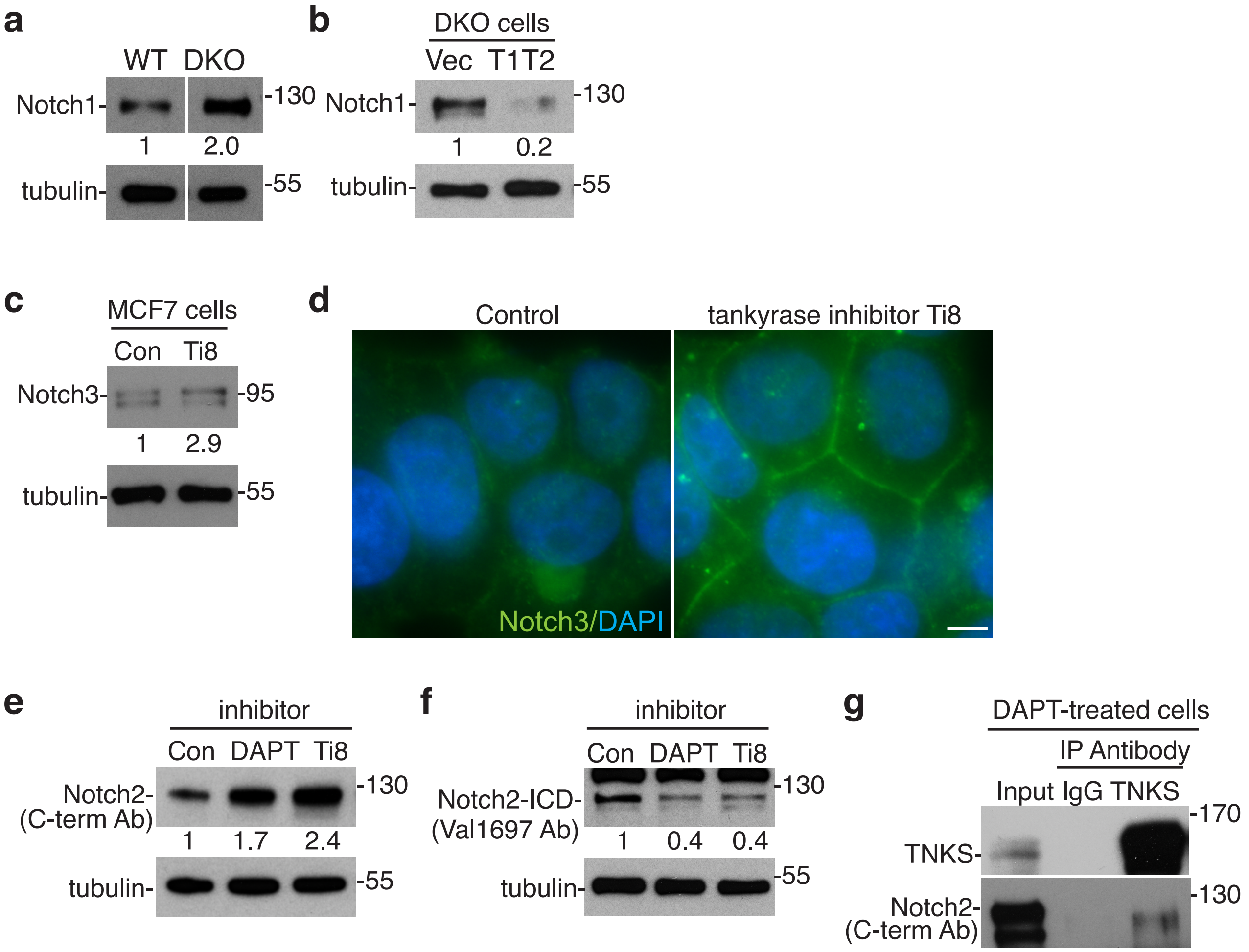

Supplementary Figure 3. Analysis of Notch1, 2, and 3. (a) and (b) Notch1 is a target of tankyrase. Immunoblot analysis of (a) WT and DKO HEK293T cells and (b) DKO cells stably expressing a Vector or T1T2 with anti-Notch1 antibody. Protein levels of Notch1 in (a) DKO versus WT or in (b) Vector versus T1T2 relative to tubulin and normalized to (a) WT or (b) Vector cells are indicated below the blot. (c) and (d) Notch3 is a target of tankyrases. (c) Immunoblot analysis of MCF7 cells treated without and with tankyrase inhibitor Ti8 and probed with anti-Notch3 antibody. Protein levels of Notch3 (upper band) in Ti8-treated cells relative to tubulin and normalized to control are indicated below the blot. (d) Immunofluorescence analysis of formaldehyde-fixed MCF7 cells treated without and with tankyrase inhibitor Ti8 and stained with anti-Notch3 antibodies (green). DNA was stained with DAPI (blue). Scale bar, 5 μm. (e) and (f) Immunoblot analysis of HEK293T cells treated without (Con) or with Ti8 or DAPT and probed with antibody that (e) detects both the cleaved and uncleaved (membrane-bound) forms of Notch2 (C-terminal antibody) or (f) detects only the cleaved form of Notch2 (Notch-ICD) (Val1697 antibody). Protein levels of Notch2 Ti8 or DAPT-treated cells relative to tubulin and normalized to control are indicated below the blot. (g) Immunoblot analysis of DAPT-treated HEK293T cells immunoprecipitated with control or TNKS IgG and probed with anti-Notch2 (C-terminal antibody).

Supplementary Figure 4 Uncropped blots

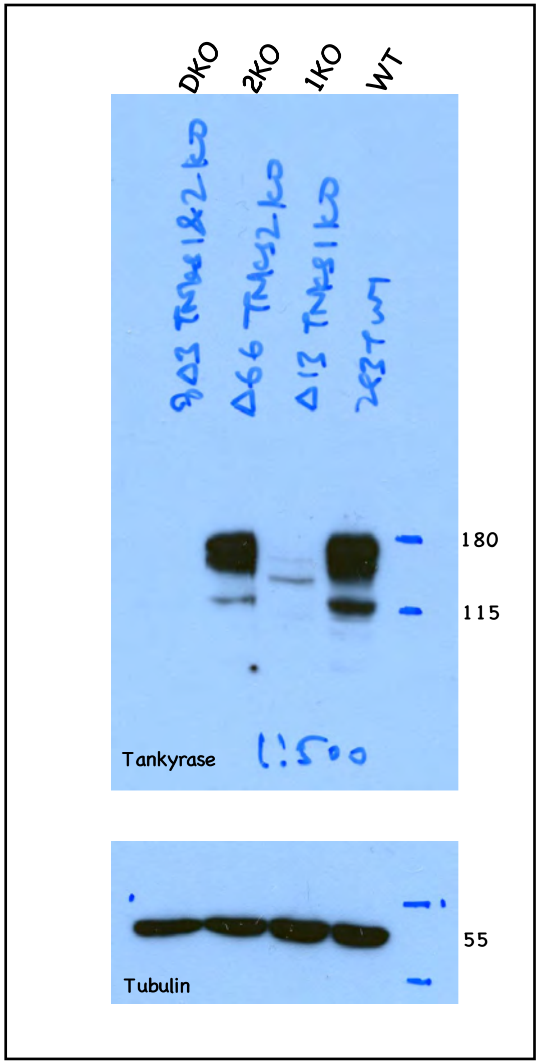

Figure 1a

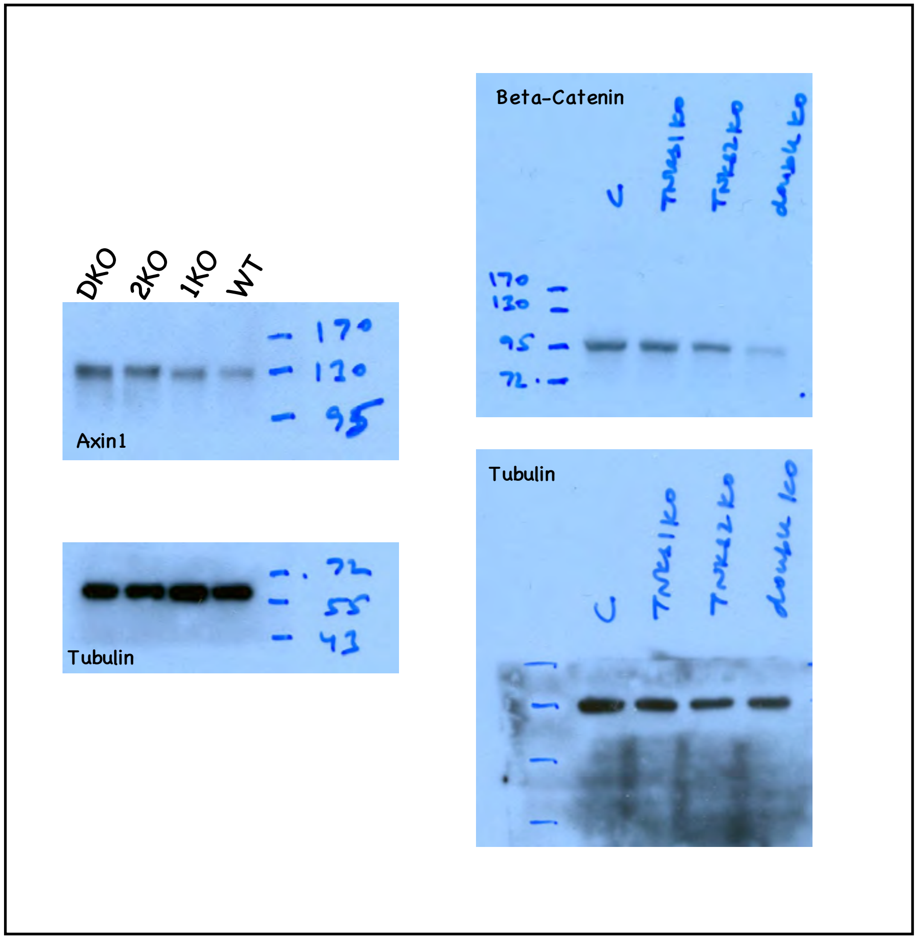
DKO
2KO
1KO
WT
170
130
95
Axin1
72
55
43
Tubulin
Beta-Catenin
170
130
95
72
Tubulin

Figure 2a

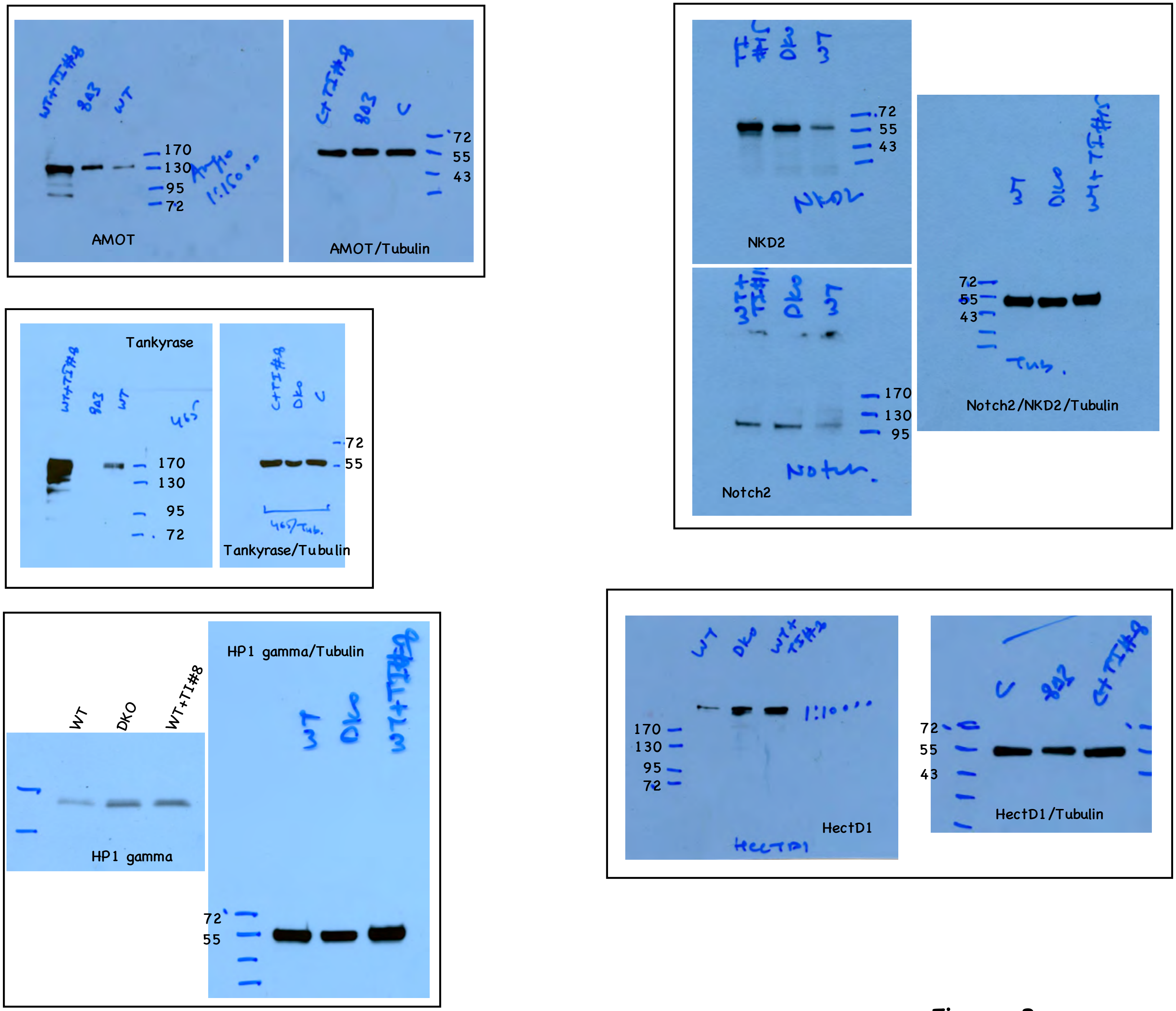
AMOT
AMOT/Tubulin
Tankyrase
Tankyrase/Tubulin
WT
DKO
WT+TI#8
HP1 gamma
HP1 gamma/Tubulin
NKD2
Notch2
Notch2/NKD2/Tubulin
HectD1
HectD1/Tubulin

Figure 3a

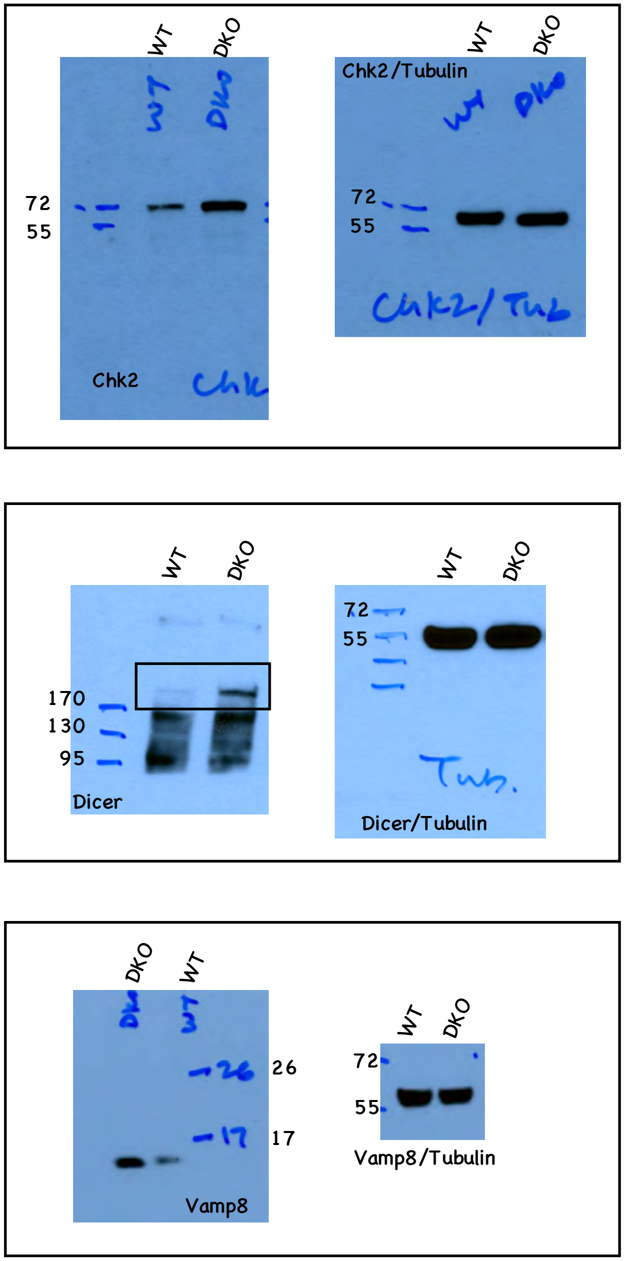

Figure 3b

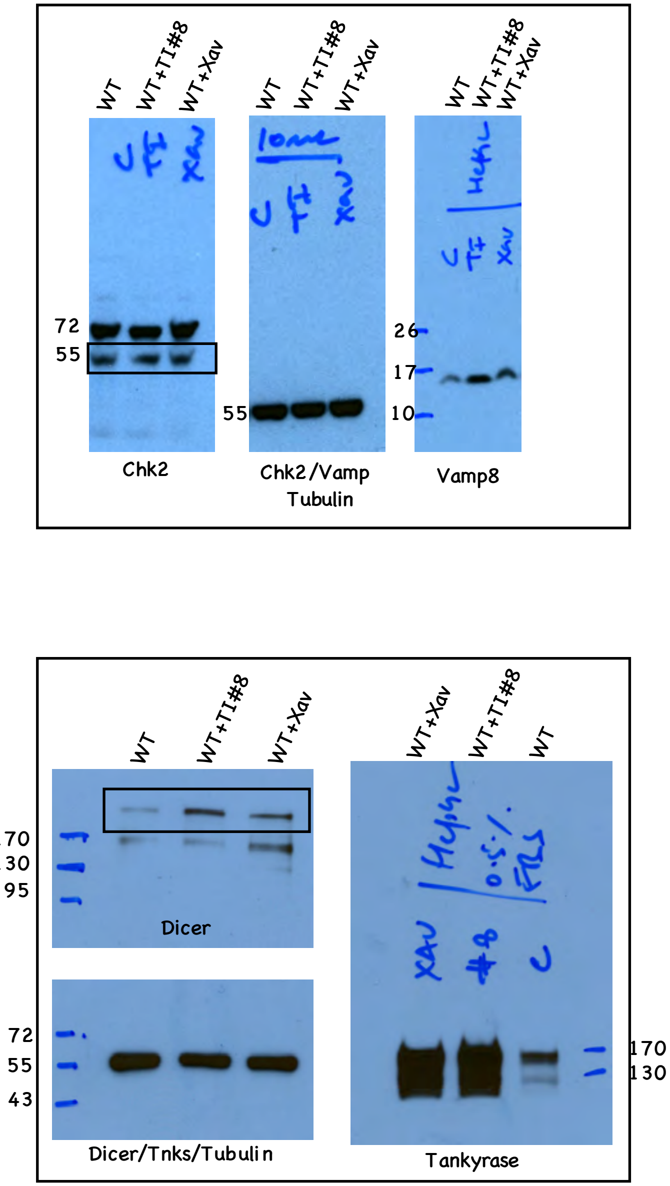

Figure 3d

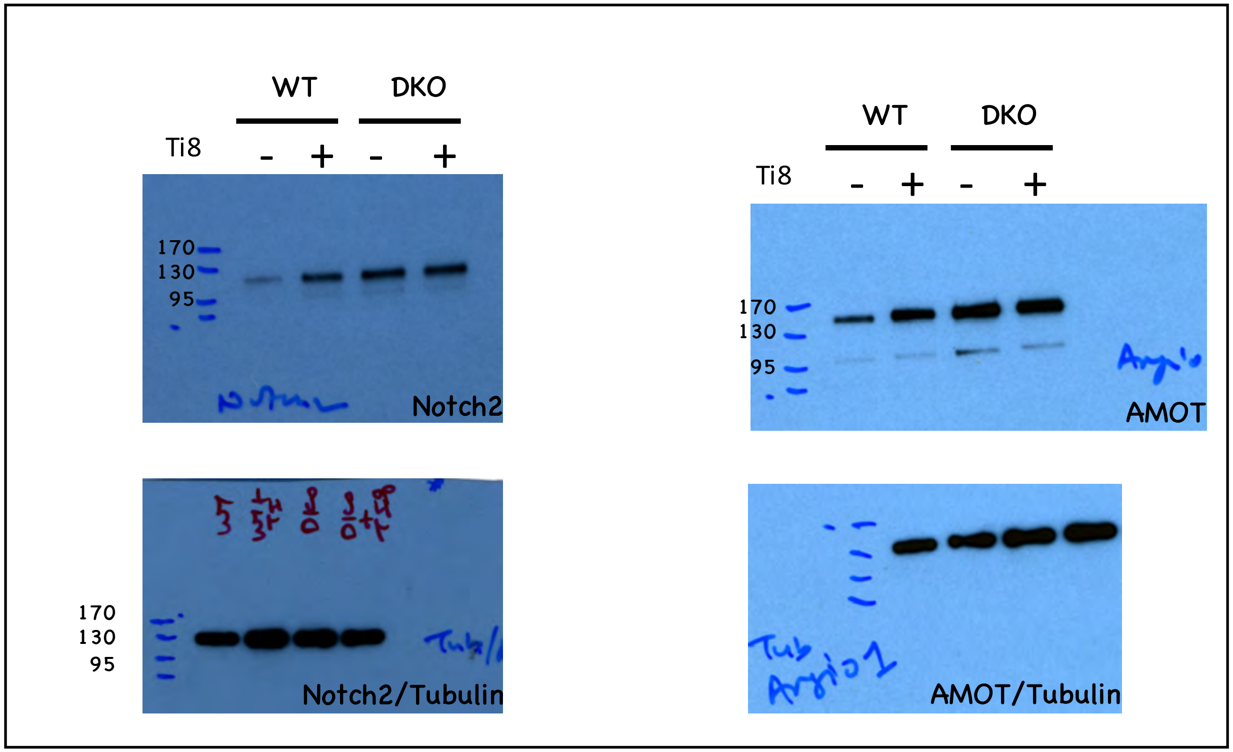
WT
DKO
Ti8
-
+
-
+
170
130
95
Notch2
170
130
95
Notch2/Tubulin
AMOT
AMOT/Tubulin

Figure 3e

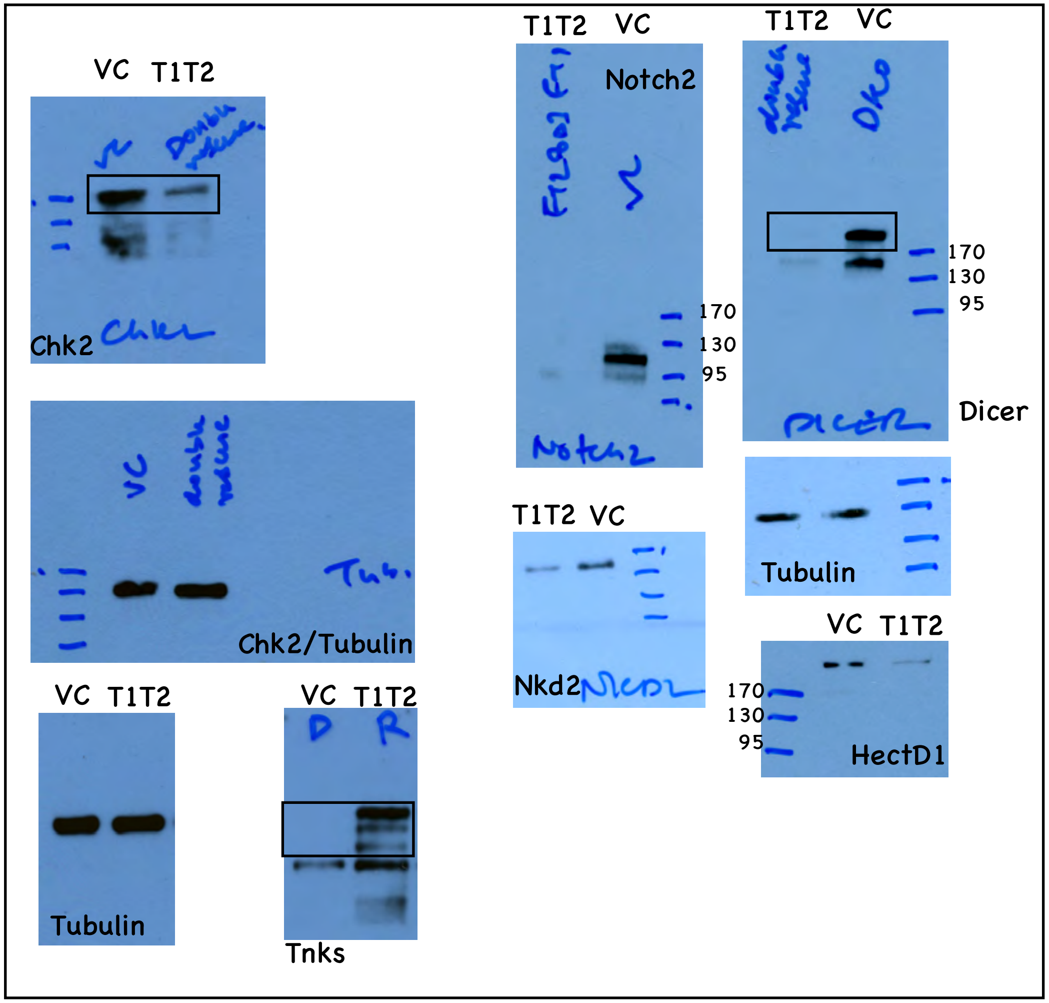

VC T1T2
Chk2
Chk2/Tubulin
VC T1T2
Tubulin
VC T1T2
Tnks
T1T2 VC
Notch2
170
130
95
T1T2 VC
170
130
95
Dicer
T1T2 VC
Nkd2
Tubulin
VC T1T2
170
130
95
HectD1

Figure 4a

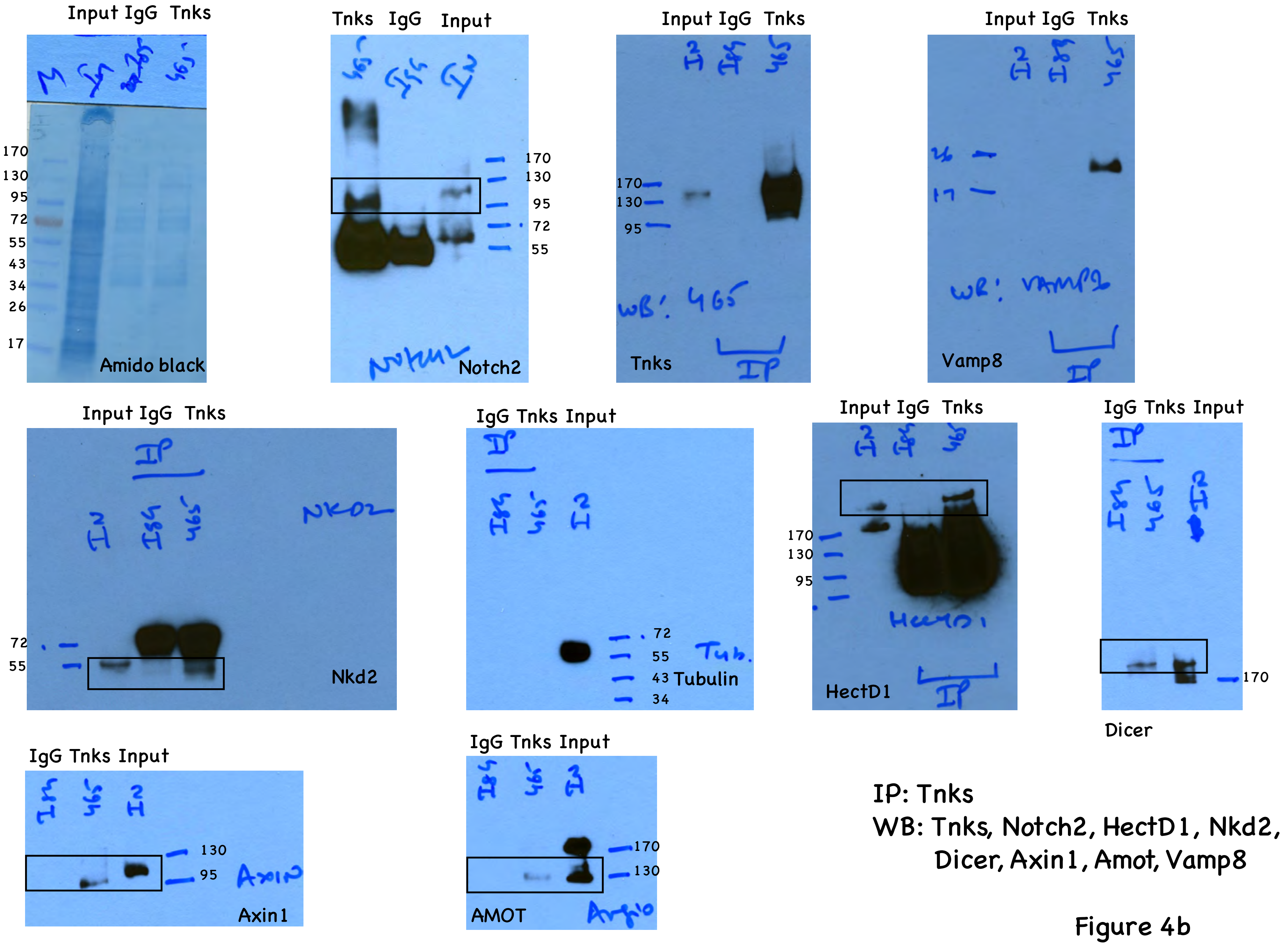

IP: Tnks
WB: Tnks, Notch2, HectD1, Nkd2, Dicer, Axin1, Amot, Vamp8

Figure 4b

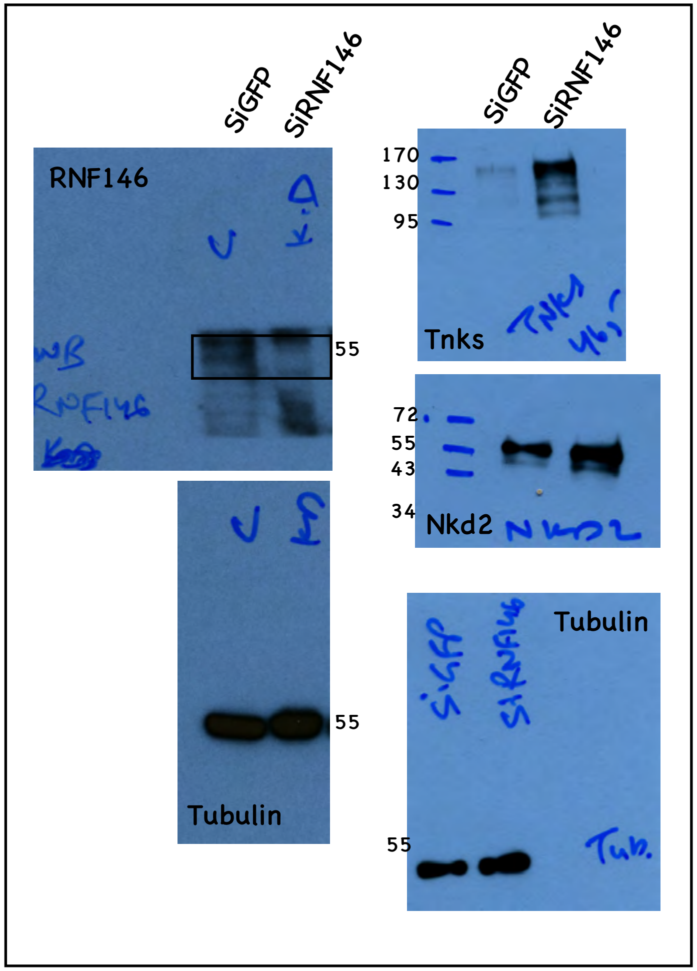

Figure 5b

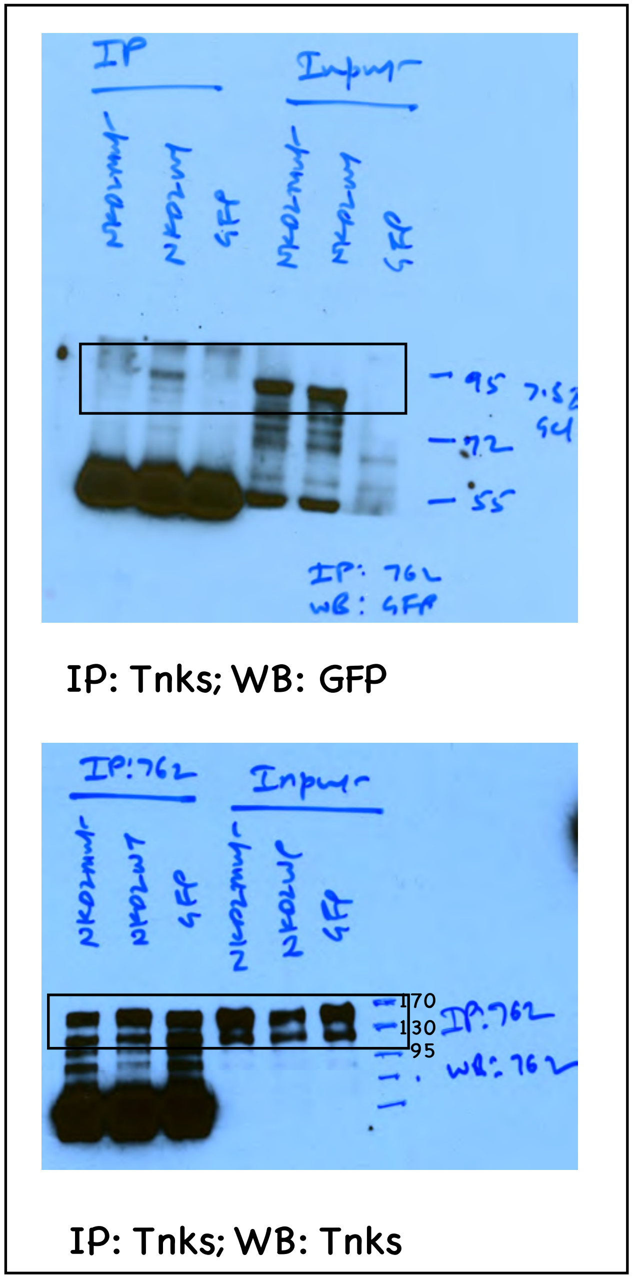

Figure 5c

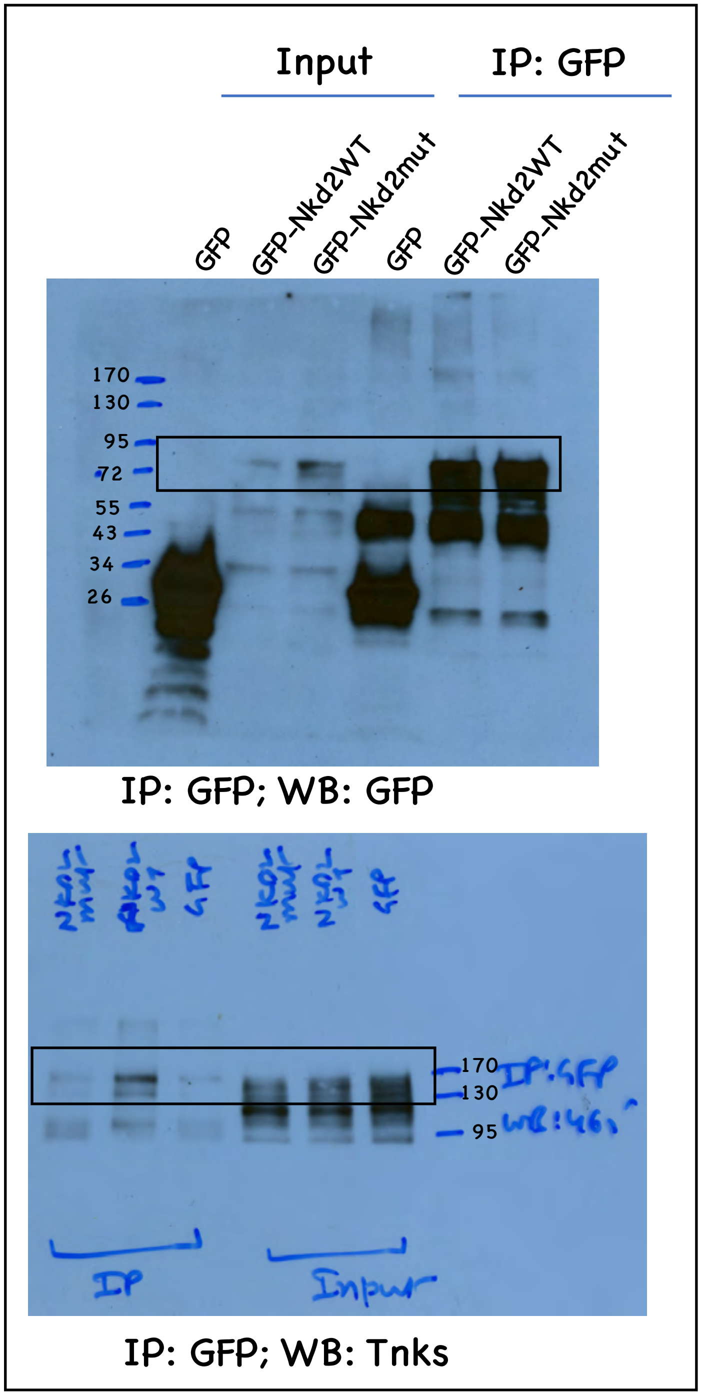

Figure 5d

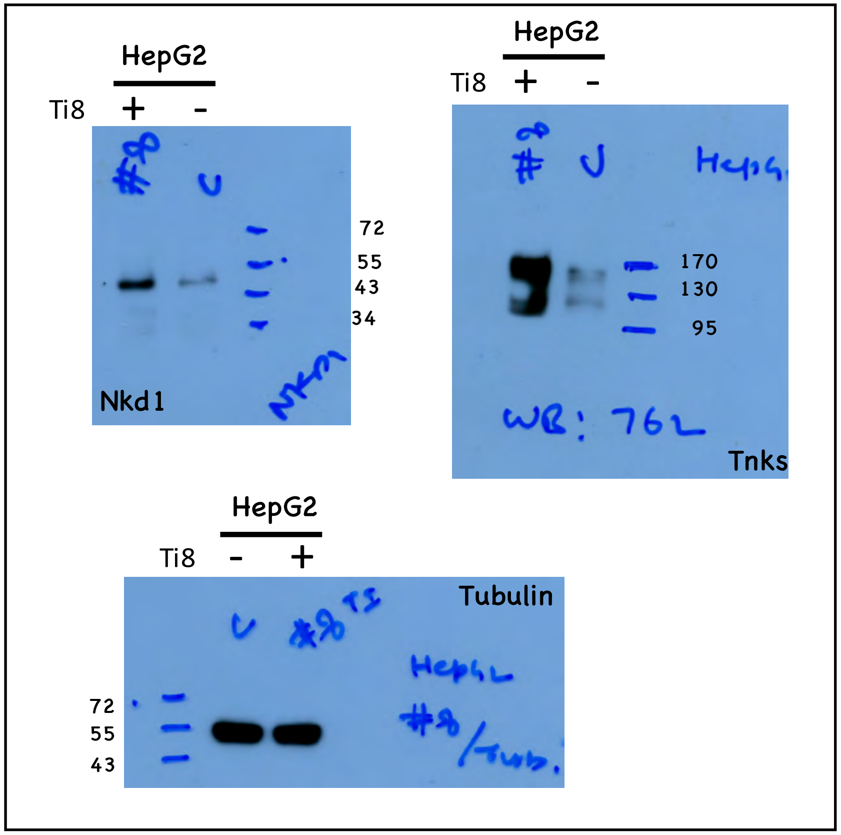

Figure 5e

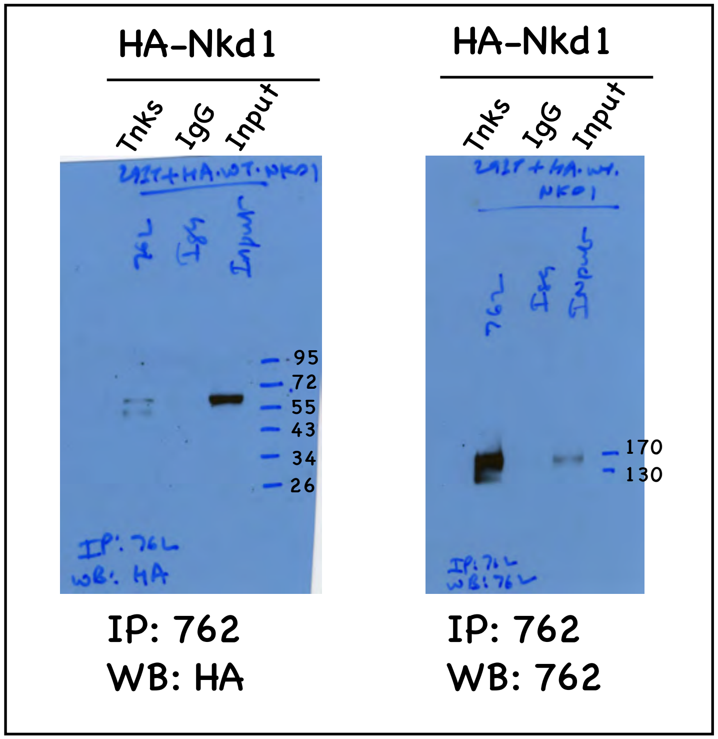
HA-Nkd1
Tnks
IgG
Input
95
72
55
43
34
26
IP: 762
WB: HA
HA-Nkd1
Tnks
IgG
Input
170
130
IP: 762
WB: 762

Figure 5f

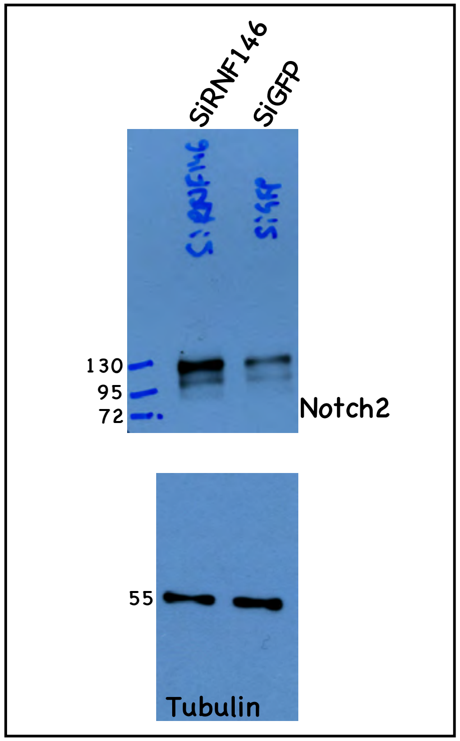

Figure 6b

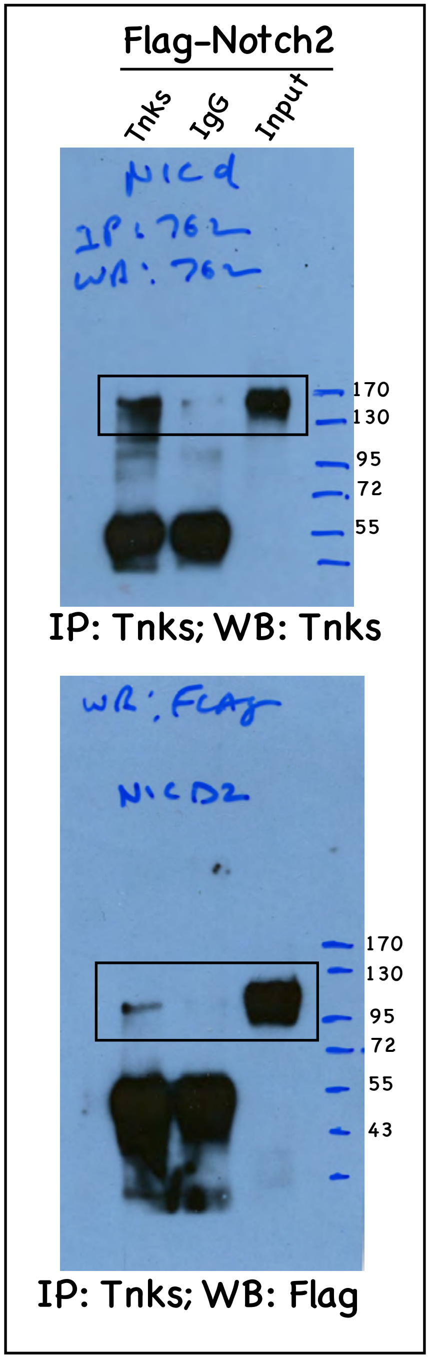

Figure 6c

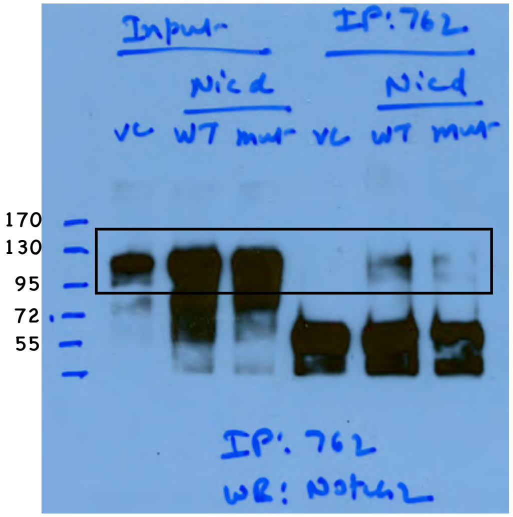

IP: Tnks; WB: Notch2

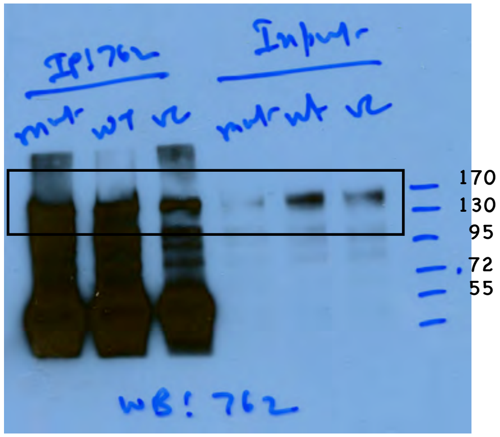

IP: Tnks; WB: Tnks

Figure 6d

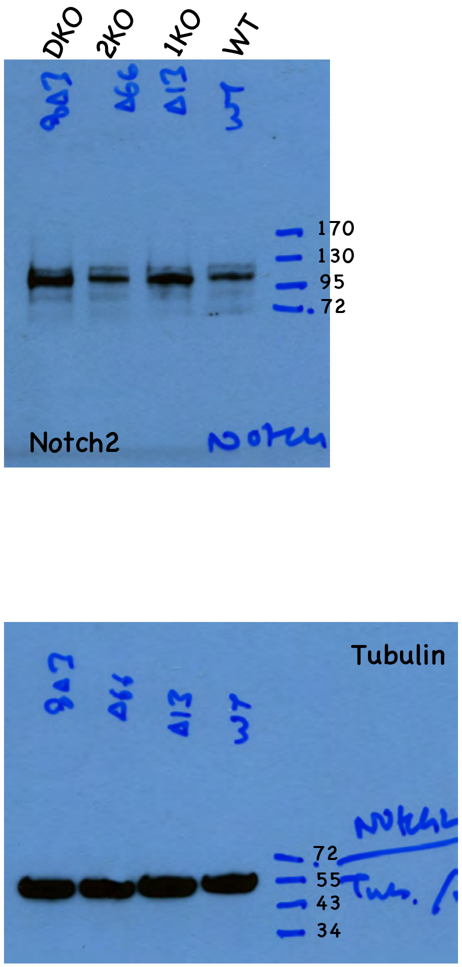

Figure 6k

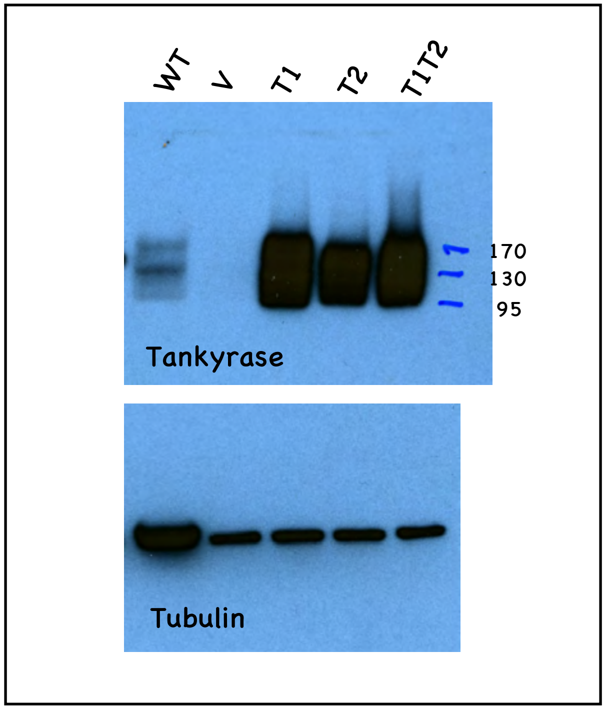

Figure S2a

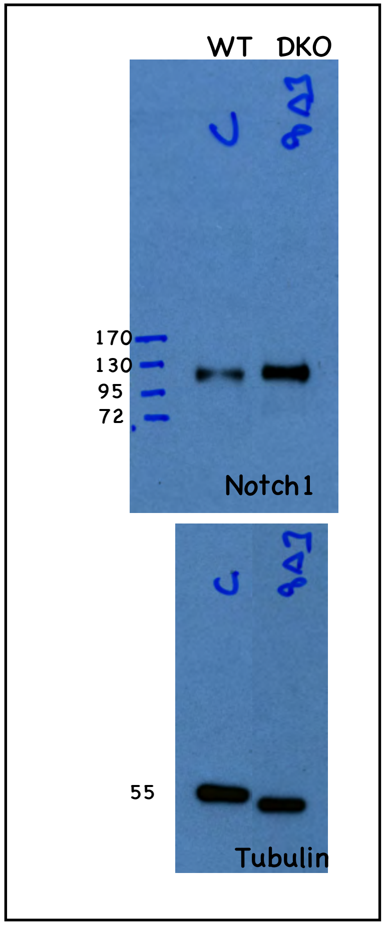

Figure S3a

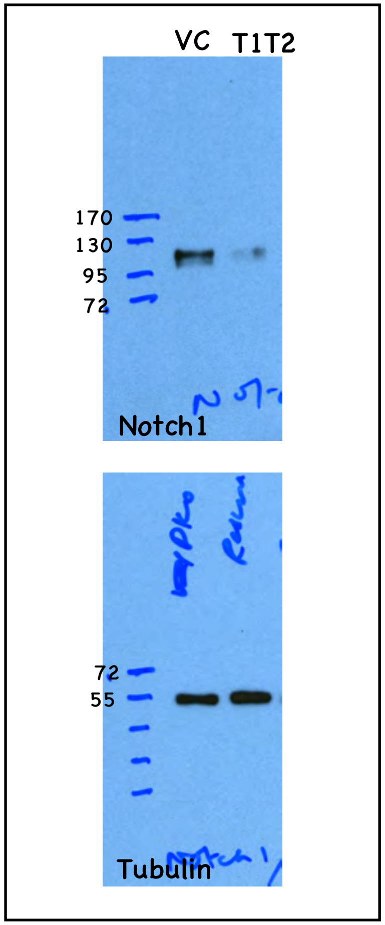

Figure S3b

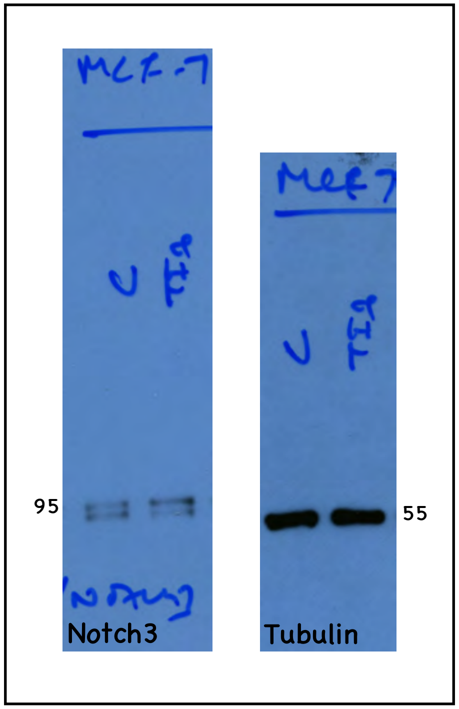

Figure S3c

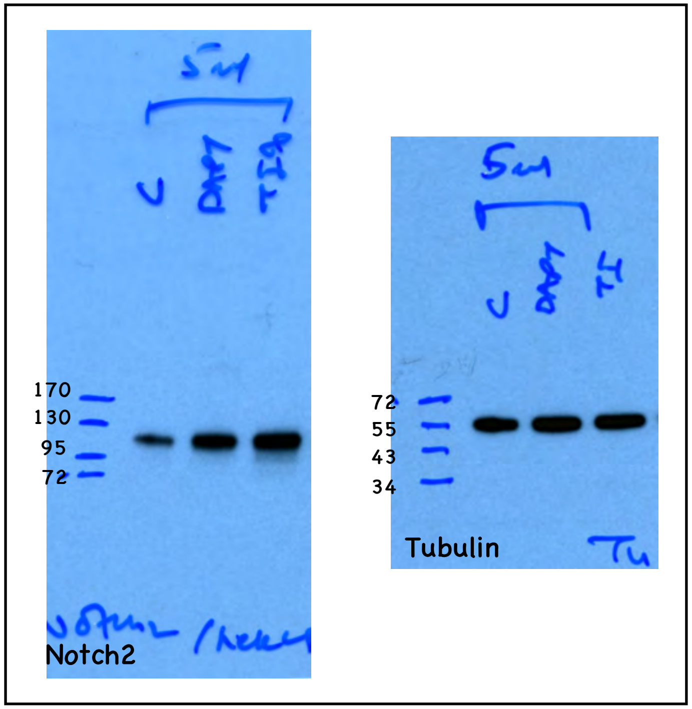

Figure S3e

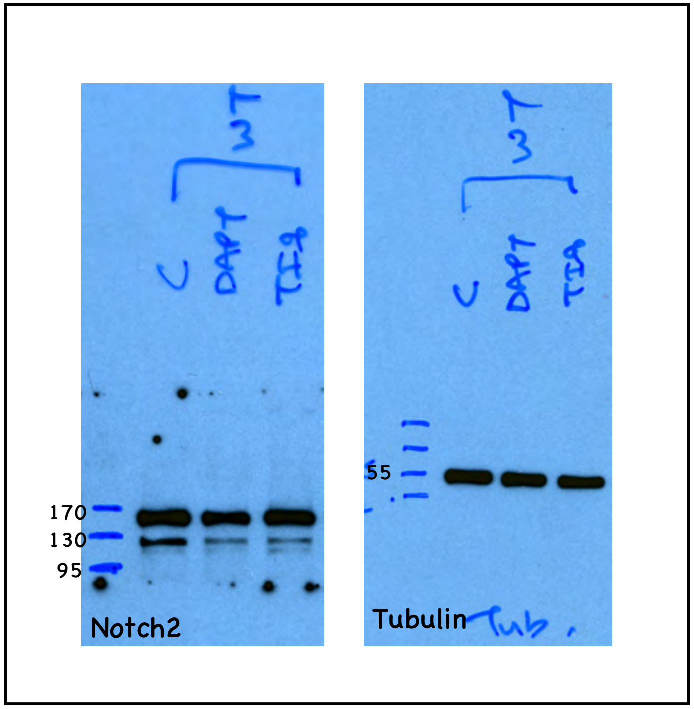

Figure S3f

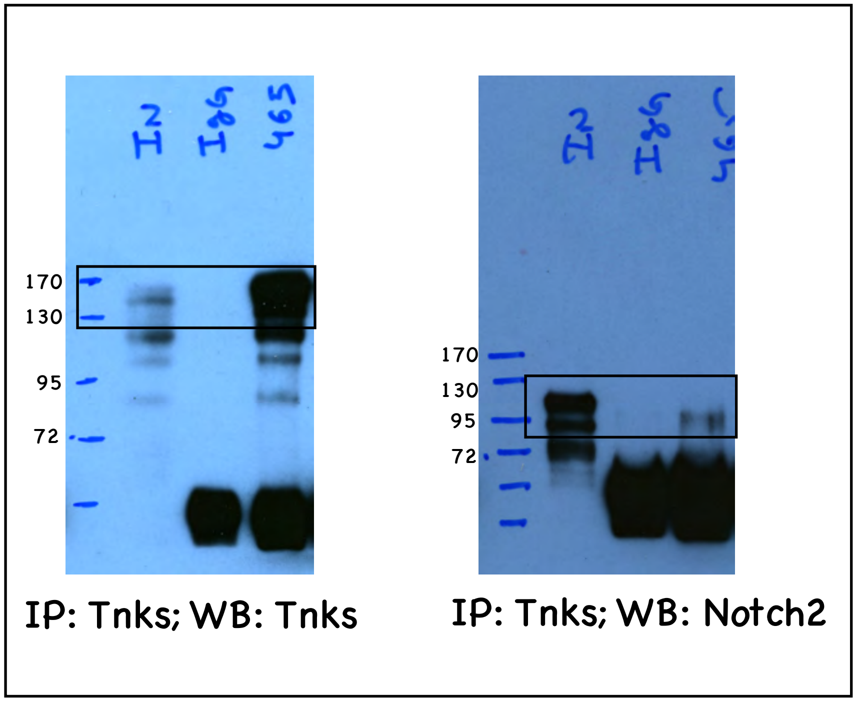

Figure S3g
