# Supplementary material for: Whole proteome analysis of human tankyrase knockout cells reveals targets of tankyrase-mediated degradation
Source: Nat Commun. 2017 Dec 20;8:2214. doi: 10.1038/s41467-017-02363-w (PMC5738441; doi:10.1038/s41467-017-02363-w)
Supplement: Supplementary file 3 — Description of Additional Supplementary Files [file 41467_2017_2363_MOESM3_ESM.pdf]

Description of Additional Supplementary Files

File Name: Supplementary Data 1
Description: List of 7254 proteins identified in the TMT analysis. Sheet 1 lists the 7254 protein groups. Sheet 2 lists the 608 (out of 7254) proteins showing a statistically significant change in abundance, indicated by (+). The gene name is indicated. Sheet 3 lists proteins with greater than 1.5-fold change in abundance. The gene name is indicated. For proteins increased in abundance the protein name and RxxG(P/A/C)xG tankyrase binding site (if present) is indicated.

File Name: Supplementary Data 2
Description: List of 74 proteins showing a significant increase in abundance and containing a RxxG(P/A/C)xG tankyrase binding site. The gene name, protein name, and tankyrase binding site is indicated.
